# Translational Medicine: Towards Gene Therapy of Marfan Syndrome

**DOI:** 10.3390/jcm11143934

**Published:** 2022-07-06

**Authors:** Klaus Kallenbach, Anca Remes, Oliver J. Müller, Rawa Arif, Marcin Zaradzki, Andreas H. Wagner

**Affiliations:** 1 Institute for Cardiac Surgery and Interventional Cardiology (INCCI), Department of Cardiac Surgery, 1210 Luxembourg, Luxembourg; kallenbach.klaus@incci.lu; 2 VASCERN HTAD European Reference Center, 1210 Luxembourg, Luxembourg; 3 Department of Internal Medicine III, University of Kiel and University Hospital Schleswig-Holstein, 24105 Kiel, Germany; anca.remes@uksh.de (A.R.); oliver.mueller@uksh.de (O.J.M.); 4 German Centre for Cardiovascular Research, Partner Site Hamburg/Kiel/Lübeck, 20251 Hamburg, Germany; 5 Department of Cardiac Surgery, University Hospital Heidelberg, 69120 Heidelberg, Germany; rawa.arif@med.uni-heidelberg.de (R.A.); marcin.zaradzki@med.uni-heidelberg.de (M.Z.); 6 Department of Cardiovascular Physiology, Heidelberg University, Im Neuenheimer Feld 326, 69120 Heidelberg, Germany

**Keywords:** marfan syndrome, aorta, gene therapy, translational therapy, aortic surgery, TGF-β

## Abstract

Marfan syndrome (MFS) is one of the most common inherited disorders of connective tissue caused by mutations of the fibrillin-1 gene (FBN1). Vascular abnormalities, such as the enlargement of the aorta with the risk of life-threatening rupture are frequently observed. However, current treatment is limited and therapeutic options focus solely on symptomatic therapy. Gene therapy focuses on genetically modifying cells to produce a therapeutic effect and may be a promising treatment option for MFS. Here, we first provide an overview of the historical background and characterization of MFS. Subsequently, we summarise current gene therapy options and possible translational concepts for this inherited disorder that affects connective tissue.

## 1. Introduction

The first descriptions of the Marfan Syndrome (MFS) by the French paediatrician Antoine Marfan in 1896 and later in the reports of others were based on skeletal features, such as joint contraction, kyphoscoliosis, hypotrophic skeletal muscles, and thin and long extremities, fingers and toes. The American ophthalmologist E. Williams described two families with a luxation of the lenses and other ocular features typical of MFS more than 20 years before Marfan. Today, we know that MFS consists of skeletal, ocular, facial (dysmorphic face), pulmonary (spontaneous pneumothorax), dermatologic (striae distensae), cardiac (mitral valve prolapses), and, especially, vascular features. The formation of aortic root aneurysms and consecutive acute aortic dissection reduces middle life expectancy significantly, and MFS is considered a devastating aortic disease.

With the development of the Berlin Nosology of Heritable Disorders of Connective Tissue Diseases in 1986, the diagnosis of MFS obtained its first uniformed structure based on clinical features [1]. In early 1990, the FBN1 gene coding for fibrillin was characterized, and it became evident that a mutation in this gene may cause MFS [2]. Therefore, the Berlin nosology was improved and replaced by the stricter Ghent nosology in 1996, where identifying an FBN1 mutation causing MFS was considered a significant criterion for diagnosing MFS [3]. Until now, the revised Ghent nosology, published in 2010, represents the gold standard for diagnosing MFS [4]. FBN1 testing holds much greater weight in the diagnostic assessment now. Next-generation sequencing (NGS) screening of the FBN1 gene and other genes possibly responsible for the hereditary aortic disease became the standard in the detection of MFS today.

Despite the tremendous improvements in understanding the molecular defects underlying MFS and the reliability of the diagnosis, the treatment of MFS has remained almost the same over the last three decades. With the introduction of the prophylactic replacement of the ascending aorta to avoid the aortic dissection type A in MFS in the late 1970s, the life expectancy of patients with MFS improved from 30–40 years to almost the anticipated average life of the unaffected population [5]. Certainly, operative standards and techniques improved. Today, a valve-sparing aortic root replacement can be operated with less than 1% operative mortality in specialized centres [6,7]. New techniques, such as the Personalized External Aortic Root Support (PEARS) appeared and arrested attention [8]. In medical drugs, the prophylactic treatment with sartanes may, in combination with beta-blockers, reduce or at least delay the development of the root aneurysm [9].

Other drugs, such as statins, are being investigated based on intensive research and improved molecular biological understanding [10]. Furthermore, the general improvement of the medical armamentarium allows for the enhanced treatment of the ocular and skeletal problems of Marfan patients. Nevertheless, Marfan syndrome remains an incisive illness for the affected patient who fears multiple operations on different organ systems, lifelong medication intake, reduced opportunities for a regular lifestyle, and possible social isolation.

Today, it is general medical knowledge that prophylaxis or even disease avoidance results in a much better outcome than treating the same disorder. Although we can sufficiently diagnose MFS and treat its complications, we cannot cause the illness to disappear. With the concept of translational medicine, we may use the vast amount of knowledge we have collected during the last decades to treat the clinical Marfan syndrome at its root—at the genetic level. With therapeutic gene concepts, it may become possible to alter the genotype of MFS so that the phenotype of the patient appears almost normal without the development of the typical complications.

At least two significant obstacles must be overcome before the successful gene therapy of Marfan syndrome becomes a clinical reality. First, the proper genetic target(s) that must be altered need to be identified. Second, a delivery method system that allows permanent expression of the target gene must be developed. We and others have previously discussed different target genes [11,12]. The TGF-β signalling pathway’s pivotal role in developing the MFS vascular phenotype may display potential target genes affecting downstream effector genes, such as Matrix Metalloproteinases (MMPs) and their inhibitors (TIMPs). The transcription factor activator protein-1 (AP-1), directly involved in TGF-β signalling and the activation of MMPs, may serve as such a target. We have shown that the neutralization of AP-1 with decoy oligodeoxynucleotides (dODN) technology reduces aortic elastolysis in an established mouse model of MFS [13]. By the modulation of the gene delivery towards RNA hairpin decoy oligonucleotide (dON)-AP1 combined with adeno-associated virus (AAV)-based transduction, we successfully aimed to improve the longevity and stability of AP-1 suppression [14]. Stimulated by these results, we here review our and others’ translational gene therapy concepts for Marfan syndrome.

## 2. Overview of Gene Therapy

Gene therapy opens an exciting new path in managing aortic aneurysm treatment and could, in the long term, be established as a novel option for Marfan syndrome. With the identification of novel therapeutic targets in maintaining endothelial and smooth muscle cell function under stress conditions [15], recent approaches have been tested in preclinical models. RNA-based therapies have recently gained considerable advances in vascular disease [16]. An overview of targets investigated in preclinical studies is listed in Table 1.

One major challenge that limits clinical translation remains the delivery method system. Although adenoviruses proved to have relatively high transduction efficiency in target cells, they are associated with critical safety concerns due to increased inflammation [17,18]. On the other hand, plasmid-based gene therapy offers inefficient and transient transduction of targets [19]. In contrast, adeno-associated viruses (AAVs) enable long-term gene expression and represent the most well-studied vector for therapeutic purposes. However, the systemic injection of AAV particles leads to low transduction efficiency in the aortic wall [20]. Local delivery methods based on stents or biocompatible materials have already been established to circumvent these obstacles, improving specificity and efficacy [21,22]. Additionally, these approaches provide reduced immunogenicity, another major challenge that prevents AAV-based strategies from being translated into clinical practice [23]. An extensive summary of the most current delivery methods, modes of application, and possibilities to increase the vascular system’s transduction efficiency have been reviewed recently [24,25].

Studies based on mouse models for Marfan syndrome brought fundamental insights into the molecular mechanisms of the pathophysiology of the disease. However, larger models are essential for further translating pharmacological and gene therapy methods into clinics. Furthermore, small animal models are inefficient for advancing surgical techniques such as local gene delivery. Moreover, due to the similar anatomy to the human subjects, treatments tested in such preclinical studies can be more readily translated into patients. A porcine model for Marfan syndrome based on an FBN1 mutation (+/Glu433AsnfsX98) has been established using genome editing and somatic cell nuclear transfer technologies [26]. The heterozygous animals present classical features described in Marfan patients, such as scoliosis, reduced bone density, and the fragmentation of elastic fibres in the aortic wall [26]. Additionally, previous naturally occurring mutations in the fibrillin-1 gene noted in calves brought pathophysiological changes similar to those observed in Marfan patients, including increased aortic dilatation and rupture and mitral valve prolapse and ectopia lenses [27,28].

In conclusion, gene therapy for Marfan syndrome remains a relatively young field. Further studies are required to improve gene transfer to the vascular system in mouse and large animal models.

## 3. Transcription Factor Decoy Technology

**Figure 1:**
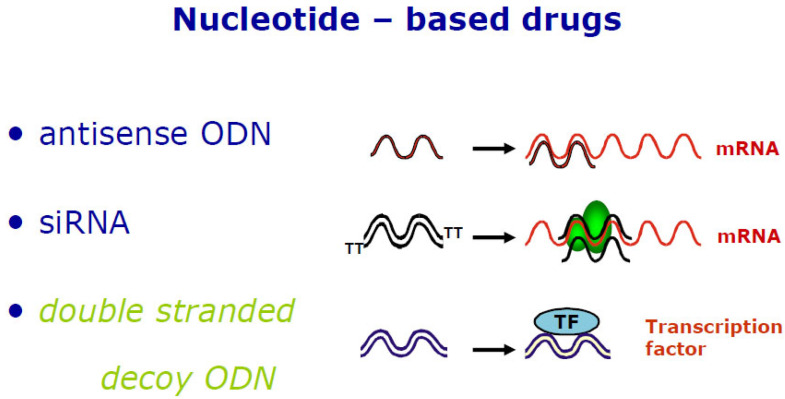
Schematic mechanism of action for antisense, siRNA, and transcription factor decoy oligodeoxynucleotides. Antisense ODNs bind to their target messenger RNA (mRNA) and inhibit or alter translation, e.g., via steric hindrance, splicing alterations, or the initiation of target degradation. The activity of small interfering RNAs (siRNA) in RNA interference (a biological process in which RNA molecules inhibit gene expression) is dependent on its binding ability to the RNA-induced silencing complex (RISC). The binding of the double-stranded siRNA to RISC is followed by unwinding and cleavage of the sense strand, which binds to its target mRNA and induces mRNA cleavage. The mechanism of the action of transcription factor decoy ODN is distinct from antisense and siRNA ODNs. Decoy ODNs are targeted to inhibit the binding of transcription factor (TF) to their cognate promoter binding sites in the genome. Reprinted from Biochem Pharmacol 144, Hecker and Wagner [39], Copyright 2017, with permission from Elsevier.

Transcription factors (TFs) are usually members of multigene families. Similar matrices represent factors within one family due to the conserved structure of DNA-binding domains [33]. Dysregulated TFs represent a promising class of drug targets that mediate aberrant gene expression, promoting various diseases. For a long time, TFs were believed to be undruggable [34]. However, advanced knowledge of protein biochemical characteristics, regulatory function, interaction with other co-factors, and the dynamics of their binding mode to DNA has paved the way for new transcription factor-targeting therapies [34]. There are several different approaches to treatment. DNA- as well as RNA-based gene silencing technologies, such as antisense oligonucleotides [35], small interfering RNAs (siRNA), or microRNA (miRNA) [36], contain specific sequences complementary to only one single target mRNA (Figure 1). This way, stability and translation are regulated and inhibited [37]. A clear disadvantage is that these approaches require a carrier for their protection and efficient delivery into the target cells [38].

Transcription factor decoy (TFD) oligodeoxynucleotides (ODN) are short double-stranded DNA molecules that contain a specific consensus transcription factor binding site (Figure 1). The binding sequence is conserved between species. It is determined by the alignment of many identified binding sites in the promoter of many target genes across different species. After cellular uptake, TFD molecules compete in the cytoplasm or nucleus with promoter regions to bind activated transcription factors [40]. This binding interferes with an aberrant expression of the disease-related genes of activated pathways based on that specific transcription factor or transcription factor family. Consensus TFD ODNs successfully tested in preclinical animal studies can be directly used in initial clinical trials to assess their safety and efficacy [39].

An advantage of the TFD technology is that the uptake of the naked ODN by most target cells does not require any transfection agents. Under physiological conditions, a carrier system and receptor-mediated endocytosis mediate the active transport [39]. A significant limitation is the rapid degradation of phosphodiester oligonucleotides by intracellular nucleases [41]. Thus, various chemical modifications have been developed to improve the nuclease resistance, long-term stability, and thermal stability [39]. Some of the drawbacks of these modifications, i.e., non-specific binding and toxicity, have led to the development of circular dumb-bell DNA molecules or hairpin TFD because of their proven resistance to exonucleases [42].

**Figure 2:**
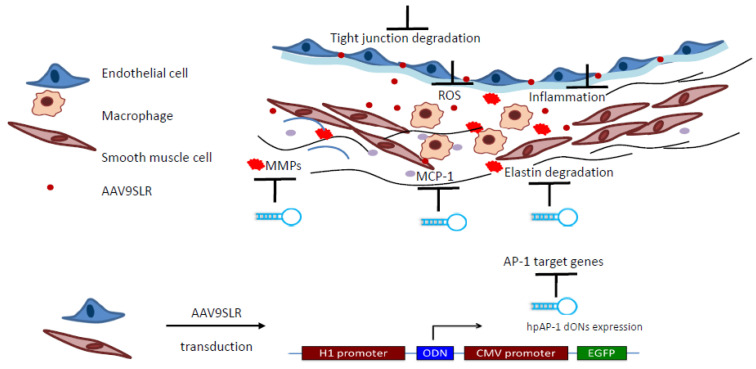
AAV-mediated AP-1 decoy oligonucleotide expression inhibits aortic elastolysis in a mouse model of Marfan syndrome. Reprinted from Cardiovasc Res 117, Remes et al. [14], Copyright 2021, with permission from Oxford University Press.

A hairpin TFD is a single-stranded homoduplex molecule like a molecular beacon. It consists of a long beacon stem containing two strands, the transcription factor binding site, and a small loop connecting them (Figure 2). The initially single-stranded ODN quickly hybridizes to itself, representing the active double-stranded TFD capable of modulating gene expression [13]. This self-hybridization property allows an intracellular single-stranded ODN vector-based expression and the formation of a functional duplex in a target cell. An adeno-associated virus (AAV)-mediated delivery and the expression of a self-hybridizing RNA TFD has been developed (Figure 2) [14,43]. RNA decoy oligonucleotides have been confirmed to bind transcription factors that generally bind DNA [44]. The safety and efficacy of AAV vectors are firmly documented in an assortment of ongoing clinical trials and at least eight human diseases [45]. Thus, AAV-based vectors are not only efficient vectors for the delivery of siRNA but also RNA TFD into dividing and nondividing mammalian cells [43].

## 4. Transcription Factor Decoy-Mediated Inhibition of Aortic Elastolysis a Fibrillin-1 Hypomorphic Marfan Mouse Model

The leading cause of the formation of aortic aneurysms in Marfan syndrome is the degradation of elastic fibres and excessive secretion of matrix-metalloproteinases (MMPs) [14]. Targeting the transcription factor activator protein-1 (AP-1) has previously been shown to offer a potential strategy for treating the vascular phenotype associated with Marfan syndrome [13,14]. What is the mechanism behind the effectiveness of this TFD approach? Fibrillin-1 renders the secreted transforming growth factor-β (TGF-β) biologically inactive by binding it to the extracellular matrix [46]. Dysfunctional fibrillin increases TGF-β bioavailability and concentration in the extracellular matrix, activating pro-inflammatory transcription factors like AP-1. The AP-1 transcription factor family regulates the MMP expression [11]. In addition, AP-1 also mediates the expression of several pro-inflammatory cytokines increased in the plasma of Marfan patients. Thus, AP-1 neutralization affects MMPs as the key enzymes in tissue remodelling. The TFD AP-1 as a possible drug candidate also can be regarded as an immune-modulator, regulating the action of genes involved in immune and pro-inflammatory responses treating the inflammatory abnormalities of connective tissue in Marfan syndrome (Figure 2).

The fibrillin-1 hypomorphic mice (mgR/mgR) are accepted as a model of Marfan syndrome and conducive to studying the clinical stages precursive to animal lethality [47]. Aortic grafts from donor Marfan mice were treated using a classical phosphorothioate-modified or hairpin DNA AP-1- TFD ex vivo. After that, they were implanted as infrarenal aortic interposition grafts and explanted 30 days later [13]. A short-term 30 min pretreatment of aortic grafts with AP-1 TFD was sufficient to significantly reduce elastolysis, macrophage infiltration, and MMP activity in the explanted grafts [13]. The integrity of the endothelial–extracellular matrix transition is lost in the aorta of Marfan mice [18] and patients [48]. Thus, the endothelial monolayer permeability was increased for TFD in the aorta of these mice with the observed loss of tight junction proteins ZO-1 and occludin, enabling the TFD to reach the tunica media [13].

A follow-up study using an RNA TFD expressing AAV in the same experimental setting described above significantly restored ZO-1 protein levels [14]. In this study, the short-term treatment and AAV transduction confirmed a stable AP-1 TFD expression in the endothelial and smooth muscle cells. Moreover, MMP expression and activity, reactive oxygen species formation, and the expression of monocyte chemoattractant protein-1 were also significantly reduced. The monocyte graft infiltration significantly declined and maintained the integrity of the elastin architecture. RNAseq analysis confirmed the beneficial effect of AP-1 neutralization on the pro-inflammatory environment in smooth muscle cells. In this study, another valuable observation was the dramatic effect of the TFD AP-1 approach to the TGF-β signalling. AAV application led to a marked decrease in TGF-β concentration in cultured human aortic smooth muscle cells from Marfan patients three days after transduction. The TFD treatment significantly decreased the downstream signalling and secreted cytokine concentration, equally as effective as applying a TGF-β neutralizing antibody in vitro.

Despite the apparent effects of the AP-1 TFD expressing viral vector, this approach cannot be transferred into clinical practice due to the ex vivo AAV application. Conventional techniques often use systemic viral vector administration. However, an injection into the blood lowers the local AAV concentration and hence the transduction efficiency, particularly within large vessels like the aorta [24]. Delivering vascular-specific AAV vectors by inserting endothelial-targeting peptides into capsid proteins might overcome this limitation [49]. In addition, a novel gene transfer approach into aortic tissue using the alginate hydrogel-mediated delivery of a retargeted AAV vector has successfully been demonstrated [22]. The AAV-containing hydrogels can be applied locally to the ascending aorta by a minimally invasive operation to bring the application closer to the clinic. Ruptures and dissections occur at this point in the aorta of Marfan mice, leading to death [47]. Future studies have demonstrated the feasibility and efficacy of this administration route.

## 5. Concepts of Others and Outlook

**Figure 3:**
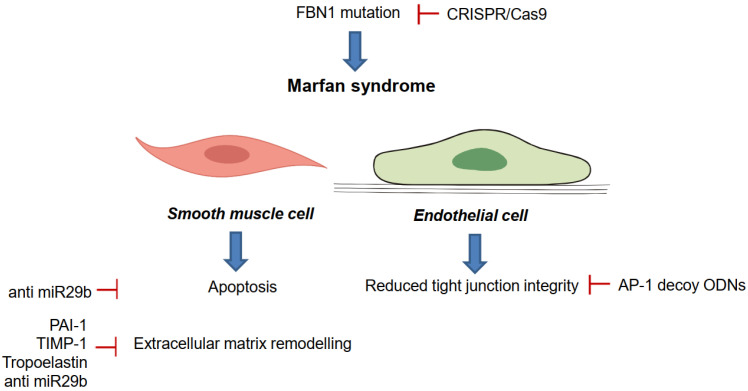
Recent concepts and preclinical approaches to treat Marfan syndrome (AP-1; activator protein-1; PAI-1, plasminogen activator inhibitor-1; TIMP-1, tissue inhibitor of metalloproteinase-1).

Over the last decade, many approaches to this genetic disorder have proven valuable in vitro and in vivo mice models. Although experimental models are widely accepted, gene therapy has not yet found its way into clinical practice. Figure 3 presents recent preclinical approaches addressed in this chapter.

The deregulation of several microRNAs (miR) is associated with developing thoracic aneurysm formation in MFS. During the early 2010s, the renowned miR 29b was identified as a critical factor in the pathogenesis of early aneurysm development in MFS by regulating aortic wall apoptosis and extracellular matrix abnormalities [50]. Almost a decade later, miR-29b suppression still constitutes a potential therapeutic target to inhibit aneurysm formation in MFS patients; however, this applies exclusively during prenatal treatment. Already existing aneurysms are not affected by the late blockade of miR29b [51]. Despite comprehensive research on miR29b as a therapeutic target, only resveratrol has been established as a drug downregulating miR29b.

Nevertheless, despite promising experimental studies on the therapeutic effects of resveratrol, a clinical investigation is lacking [52]. Interestingly, beneficial theoretical effects on Marfan cardiomyopathy and endothelial dysfunction by oxidative stress changes in miR21/miR29 balance support resveratrol’s clinical research [53]. Other players in the miR family were also deregulated in the peripheral blood and the aortic wall of Marfan patients [54]. Most of those will probably share miR29 b’s fate and will not be able to change the outcome of Marfan aortopathy.

Another possible target is the extracellular matrix (ECM) marker plasminogen activator inhibitor PAI-1. The overexpression of PAI-1 prevented the development of abdominal aortic aneurysms in mice. However, despite promising experimental results, this approach has not been further investigated [55]. Similarly, the overexpression of TIMP aiming to restore the balance within the ECM by reducing the burden of matrix metalloproteinases in the aortic media was not as compelling as once thought [29]. In a murine heterotopic aortic transplant model (mgR/mgR mice), the adenovirus-induced overexpression of TIMP-1 led to massive intima hyperplasia in the Marfan aorta [18]. However, TIMP-1 overexpression significantly reduced MMP9-activity in a heterotopic aortic transplant model when delivered by apathogenic AAVs [38]. As AAV transduction did not result in intima hyperplasia in Marfan aortic grafts [14], it represents a possible beneficial alternative gene therapy approach to adenovirus. Adenovirus-mediated overexpression of tropoelastin aimed to induce the production of healthy elastin fibres to prevent further damage to the aortic wall [30] and proved helpful in rats. However, this approach was not pursued in clinical research. Nevertheless, AAV-mediated gene therapy has several advantages for MFS. Most importantly, the local treatment of the ascending aorta seems feasible without taking the risk of systemic reactions [22].

Several gene therapeutic approaches have been established and investigated in animal models to achieve a unique therapeutic possibility for MFS. Most of those approaches at least had the benefit of elucidating a particular part of the pathogenic mechanism toward a better understanding of MFS. Yet, an unanswered question remains for many vector-mediated therapeutic approaches: How will they be transferred into clinical practice? Our working group recently proved a transadventitial uptake of AAV-mediated EGFP into the aortic smooth muscle and endothelial cells without adverse reaction in terms of affecting other solid organs by our SMC and endothelial cell-specific AAV [22]. Moreover, the increasingly comprehensive Personalized External Aortic Root Support (PEARS) technique is a feasible alternative to conventional root preserving techniques. Interestingly, the histological analysis of an MFS patient who underwent PEARS showed complete incorporation of the microporous mesh into the aortic adventitia [56]. Our local AAV therapy and incorporating the PEARS mesh (cave: *n* = 1) demonstrate possible methods for a comprehensive therapy of the ascending aorta if gene therapy is combined or implemented in absorbable or amicable fabrics.

Multiple sequencing analyses have brightened the rather grim outlook in recent years. Many novel therapeutic targets, RNAs, and DNA alterations were identified and described and multi-omics and single-cell sequencing revealed novel pathways and targets [57,58,59]. Using inducible pluripotent stem cells derived from MFS patients delivered more insights into possible genetic targets [60]. The sequencing methods mentioned above have great potential to be transferred into state-of-the-art research [61]. The phenotype-to-genotype correlation is essential for future therapies as not every genotype results in a life-threatening phenotype in MFS. Arnaud et al. have shown that in-frame mutations could be subdivided according to their impact on the cysteine content of fibrillin-1, with a global higher severity and aortic risk for cysteine loss variants and the highest frequency of ectopia lentis surgery for cysteine addition variants [62]. Mutations resulting in haploinsufficiency resulted in more cases of aortic dissection and occurred at a younger age [63]. Thus, the phenotype-to-genotype correlation can be used for optimal risk stratification of patients, with great importance for genetic counselling and personalized medicine [62].

Identifying crucial life-threatening novel mutations is the only way to potentially implement CRISPR/Cas9 therapy, the ultimate objective for causal gene therapy for MFS with high aortic risk. Establishing a wide range of shared gene databases needs to be one of the primary goals of dedicated Marfan centres to identify potential new mechanistic targets. This aim was already brought forward almost 15 years ago in the form of a bioinformatic network to correlate genotype and phenotype [64]. Still, it needs further competition, especially in light of recent ground-breaking developments in gene-editing technology. CRISPR/Cas9 is a relatively new gene alteration approach, established using bacterial enzymes and by “reprogramming”, creating a fast and easy way for gene editing.

CRISPR/Cas9 creates specific double-strand breaks that trigger DNA repair mechanisms at the target locus. CRISPR/Cas9 consists of the endonuclease Cas9 and a noncoding guide RNA. The guide RNA binds to complementary DNA. When pairing to the target sequence, the Cas9 endonuclease induces specific double-stranded breaks (DSBs), which are repaired by cellular DNA damage response machinery. Thus, by generating the cellular repair machinery and replacing the mutated DNA sequence, CRISPR/Cas9 can be used to delete, replace, or add genetic sequences [65]. This gene-editing approach can be beneficial for monogenetic gene disorders. One needs the database mentioned above to successfully edit those genes in question, which can only be generated through the consequent translation of clinical phenotypes into genotypes. The potential advantage of gene editing for FBN-1 mutation has been described by Zeng and colleagues [66]. The proof of concept of embryonic gene editing was established earlier. In 2017, MYC3, one of the responsible transcription factors for developing hypertrophic cardiomyopathy, was first targeted in skin-derived pluripotent stem cells and later in human embryos. The MYBPC3∆GAGT deletion was successfully corrected using CRISPR/Cas9 [67]. In the case of the FBN-1 mutation correction, the T7498C mutation was targeted and successfully eliminated using the approach above. The treated human embryos were tested, and no modification in the previously affected allele was found.

In conclusion, basic research is necessary to understand and elucidate the pathomechanisms of MFS and to find promising targets or mechanisms of action that can be tackled by either existing or new drug formulations. These investigations are most important for patients who have already developed the disease and require better medication and treatment options. In addition, contribution to a gene database for confirmed MFS mutations will be more critical than ever if gene correction using CRISPR/Cas9 emerges as a clinically applicable method.

## Figures and Tables

**Table 1:** Known targets in the gene therapy of aneurysm.

Target	Animal Model	Reference
TIMP-2	Elastase perfusion (rat)	[29]
Tropoelastin	Elastase perfusion (rat)	[30]
ACE2	AngII infusion (mouse)	[31]
TGF-β	Marfan syndrome (mouse)	[32]

## List of Abbreviations

AAV	adeno-associated virus
AP-1	activator protein-1
dODN	decoy oligodeoxynucleotide
dON	decoy oligonucleotide
FBN1	fibrillin-1
MMPs	Matrix Metalloproteinases
MFS	Marfan syndrome
PAI-1	plasminogen activator inhibitor-1
TFs	transcription factors
TFD	transcription factor decoy
TGF-β	transforming growth factor-β
TIMP-1	tissue inhibitor of metalloproteinase-1
